# Through the looking glass: CNS imaging findings in HAM/TSP

**DOI:** 10.1186/1742-4690-11-S1-O29

**Published:** 2014-01-07

**Authors:** Raya Massoud

**Affiliations:** 1 Viral Immunology Section, Neuroimmunology Branch, National Institutes of Neurological Disorders and Stroke, Bethesda, MD, USA

## 

No abstract submitted

